# Subcutaneous Foslevodopa as Novel Rescue Therapy for Akinetic Crisis in Parkinson's Disease: A Case Report

**DOI:** 10.1002/mdc3.70138

**Published:** 2025-05-16

**Authors:** Bendix Labeit, Julius Schulten, Alfons Schnitzler, Christian Johannes Hartmann

**Affiliations:** ^1^ Medical Faculty and University Hospital Düsseldorf, Department of Neurology Heinrich‐Heine‐University Düsseldorf Düsseldorf Germany; ^2^ Institute of Clinical Neuroscience and Medical Psychology, Medical Faculty Heinrich‐Heine University Düsseldorf Germany

**Keywords:** Akinetic crisis, Parkinson's disease, subcutaneous foslevodopa

In Parkinson's disease (PD), an akinetic crisis represents a severe, life‐threatening complication characterized by a sudden, profound loss of motor functions, including severe dysphagia, which impedes the ability to swallow oral medications. Such crises are frequently triggered by infections, surgery, or interruptions in regular medication regimens, leading to complete immobility. This immobility significantly raises the risk of serious complications such as aspiration, pneumonia, deep vein thrombosis and pulmonary embolism, all of which can be fatal. Managing an akinetic crisis thus poses a significant challenge in clinical settings. Common treatment strategies include enteral levodopa applications, apomorphine injections, dopamine agonist patches, and intravenous amantadine, supplemented by intensive care monitoring, and meticulous fluid and electrolyte management.^1^

From a pathophysiological perspective, subcutaneous foslevodopa could serve as an ideal rescue therapy for akinetic crises, potentially offering a valuable complement to traditional treatments. Recently approved, foslevodopa, a water‐soluble phosphorylated prodrug of levodopa, is designed for continuous subcutaneous injection to manage motor fluctuations,^2^, ^3^ which are commonly seen as PD progresses. Despite emerging evidence supporting its effectiveness in improving motor fluctuations, data on the use of subcutaneous foslevodopa in akinetic crises remain scarce. This report explores its effective off‐label application in a PD patient, aiming to offer insights into its potential benefits and expand the understanding of its therapeutic scope in acute settings.

## Case Report

A 66‐year‐old male with a 13‐year history of PD, hypokinetic‐rigid type, Hoehn and Yahr Stage 3, underwent uncomplicated left knee arthroplasty at an external clinic. He self‐discharged prematurely against medical advice the day after surgery and developed progressive wound dehiscence and a periprosthetic infection. Three weeks post‐operation, he was readmitted via the emergency department to the initial hospital. Due to a critical oversight, the patient's established dopaminergic medication regimen was not resumed after transfer. He had been taking levodopa/benserazid 100/25 mg eight times daily, levodopa/benserazid extended‐release 200 mg, and levodopa/benserazid rapid‐release 100 mg, with a total daily levodopa equivalent dosage of 1100 mg. The omission of these medications led to an akinetic crisis, necessitating an intensive care unit transfer to our facility.

Upon ICU admission, he was soporous (Glasgow Goma Sacle of 8), feverish (38.2°C) and showed a mildly elevated CPK level of 247 U/L (reference <171 U/L), suggesting a possible overlap with Parkinsonian hyperpyrexia syndrome. We reinitiated his dopaminergic therapy via nasogastric tube, improving his vigilance within a day, though severe dysphagia persisted. His motor functions were persistently impaired, with a Movement Disorder Society‐sponsored Unified Parkinson's Disease Rating Scale (MDS‐UPDRS‐III) of 89 points. Following repeated unsuccessful attempts to reinsert the nasogastric tube, we initiated subcutaneous foslevodopa/foscarbidopa therapy. The infusion was administered at a rate of 0.42 mL/h from 6:00 a.m. to 10:00 p.m., and reduced to 0.21 mL/h overnight. The concentration of the infusion was 240 mg/12 mg per mL, resulting in daily doses of 2034 mg of foslevodopa and 101 mg of foscarbidopa. Within 3 days, this treatment markedly improved the patient's motor functions, reducing the MDS‐UPDRS‐III score to 34. Additionally, it normalized the patient's vigilance and resolved the fever. Once his swallowing function returned, oral medications were reintroduced. He later underwent a successful revision of the knee prosthesis.

## Discussion

This case report highlights the potential utility of subcutaneous foslevodopa treatment as an off‐label rescue therapy for akinetic crises, especially in situations where enteral administration is either impractical or fails. Our findings are in agreement with those reported by Loeffler et al,^4^ who observed significant improvement in kinetics after initiating subcutaneous foslevodopa in a patient with normal pressure hydrocephalus during a pulmonary infection. Notably, their patient was dopamine‐naive prior to treatment. Similarly, our case demonstrates that subcutaneous foslevodopa could be an effective rescue option for managing akinetic crises in patients with PD.

It is important to underscore that the use of subcutaneous foslevodopa to address an akinetic crisis is currently off‐label. Our observations underscore the imperative for rigorous future studies to explore and validate the efficacy and safety of subcutaneous foslevodopa treatments in akinetic crises, including comparisons with other therapeutic modalities. Future research could explore the rapid onset of this treatment and its potential side effects, such as cognitive issues or psychosis. These effects may differ from those seen with the seamless substitution of oral medications during clinically stable phases in treatments for chronic motor complications. Foslevodopa may also be considered as an acute substitute in PD patients with gastrointestinal dysfunction, potentially to prevent the development of akinetic crisis; however, further reports and studies are needed to establish its efficacy in this context. Furthermore, practical challenges that need addressing include the lack of pumps designed for non‐patient‐specific use and the absence of data on the equivalency of standard clinical perfusors as alternatives in emergency settings.

Despite these challenges, this case underscores the promising potential of subcutaneous foslevodopa therapy in managing akinetic crises in PD. We advocate for further investigation into its safety, efficacy, and the operational logistics required to integrate it into clinical practice effectively.

## Author Roles

(1) Research project: A. Conception, B. Organization, C. Execution; (2) Statistical Analysis: A. Design, B. Execution, C. Review and Critique; (3) Manuscript Preparation: A. Writing of the first draft, B. Review and Critique.

B.L.: 1A, 1B, 1C, 3A, 3B.

J.S.: 1C, 3B.

A.S.: 1A, 1B, 1C, 3A, 3B.

C.H.: 1A, 1B, 1C, 3A, 3B.

## Disclosures

**Ethical Compliance Statement:** The authors confirm that institutional review board approval was not required for this study. Additionally, we confirm that we have read the Journal's position on the issues involved in ethical publication and affirm that this work is consistent with these guidelines. We explained the use of patient information in our article and obtained written informed consent.

**Funding Sources and Conflict of Interest:** No specific funding was received for this work. The authors declare no conflicts of interest linked to this manuscript.

**Financial Disclosures for the Previous 12 Months:** Unrelated to this work. AS has received research funding by the Deutsche Forschungsgemeinschaft (DFG), and has received consulting fees and/or speaker honoraria from Abbott, Abbvie, Alexion, bsh medical communication, Kyowa Kirin, Novartis, and Zambon. BL has received research funding from the German Research Foundation (DFG), Clexio Bioscience Ltd, the Medical Faculty of the University of Münster, has received scientific prizes from the German Geriatric Society (DGG) in conjunction with the Rolf and Hubertine Schiffbauer Foundation and the German Neurological Society (DGN), has received honoraria and travel grants for lectures and conferences from the German Society for Clinical Neurophysiology and Functional Imaging (DGKN), the European Stroke Organization (ESO), the Society for Clinical Nutrition and Metabolism (ESPEN), Phagenesis Ltd, the German Society for Neurology (DGN), the European Society for Swallowing Disorders (ESSD), the United European Gastroenterology (UEG), the University of Strasbourg, the German Society for Muscular Dystrophy (DGM), the German Society for Nutritional Medicine (DGEM), the German Society for Internal Medicine (DGIM), the University of Zurich, the Confederation of European Otorhinolaryngology—Head and Neck Surgery (CEORL‐HNS), Novartis, and Danone. The other authors declared no conflicts of interest. CJH has received honoraria from Abbvie, Abbott, Alexion Pharma GmbH Germany, Orphalan, and Univar solutions.

## Data Availability

Data sharing is not applicable to this article as no new data were created or analyzed in this study.
